# Comparison of Clinical Features in HLA-B27 Positive and Negative Patients With Axial Spondyloarthritis: Results From a Cohort of 4,131 Patients

**DOI:** 10.3389/fmed.2020.609562

**Published:** 2020-12-23

**Authors:** Shangzhu Zhang, Yanhong Wang, Linyi Peng, Jinmei Su, Xiaofeng Zeng, Mengtao Li, Zhenbiao Wu, Jian Xu, Min Yang, Lijun Wu, Cheng Zhao, Xinwang Duan, Qin Li, Jing Zhu, Wenqiang Fan

**Affiliations:** ^1^Key Laboratory of Rheumatology and Clinical Immunology, Ministry of Education, Ministry of Science and Technology, Department of Rheumatology, National Clinical Research Center for Dermatologic and Immunologic Diseases, Peking Union Medical College, Peking Union Medical College Hospital, Chinese Academy of Medical Sciences, Beijing, China; ^2^Department of Epidemiology and Bio-Statistics (YW), Peking Union Medical College, Institute of Basic Medical Sciences, China Academy of Medical Sciences, Beijing, China; ^3^Department of Rheumatology, Xijing Hospital affiliated to the Fourth Military Medical University, Xi'an, China; ^4^Department of Rheumatology, Kunming Medical University First Affiliated Hospital, Kunming, China; ^5^Department of Rheumatology, Nanfang Hospital Affiliated to Southern Medical University, Guangzhou, China; ^6^Department of Rheumatology, The People's Hospital of Xinjiang Uygur Autonomous Region, Urumchi, China; ^7^Department of Rheumatology, Guangxi Medical University First Affiliated Hospital, Nanning, China; ^8^Department of Rheumatology, Nanchang University Second Affiliated Hospital, Nanchang, China; ^9^Department of Rheumatology, First People's Hospital of Yunnan, Kunming, China; ^10^Department of Rheumatology, Sichuan Provincial People's Hospital, Chengdu, China; ^11^Department of Rheumatology, Xinxiang Central Hospital, Xinxiang, China

**Keywords:** HLA-B27, family history, uveitis, psoriasis, axial spondyloarthritis (axSpA)

## Abstract

**Objective:** The aim of our study was to assess the influence of the HLA-B27 status on axial spondyloarthritis (axSpA) in the largest cohort in China.

**Methods:** An observational, cross-sectional, and analytic study of axSpA patients from the China axSpA database was performed. Demographic and clinical data were compared in terms of the HLA-B27 status. Univariate and multivariate analyses were performed to identify variables related to HLA-B27 presence.

**Results:** We enrolled 4,131 patients in this study; of those, 36,95 (89.4%) were HLA-B27 positive. In the multivariate analysis, male gender (*p* < 0.001), younger age (*p* < 0.001), a disease duration of more than 3 years (*p* < 0.001), a family history of SpA (*p* < 0.001), uveitis (*p* < 0.001), ASDAS-CRP (*p* < 0.001), and biologic treatment (*p* < 0.001) were the main variables that were independently related to HLA-B27 presence, whereas a diagnosis delay time >36 months (*p* < 0.001) and psoriasis (*p* < 0.001) were independently related to HLA-B27 absence.

**Conclusion:** In Chinese axial SpA patients, presence of HLA-B27 is associated with the male sex, younger age, longer disease duration, greater family aggregation, and higher frequency of uveitis; absence of HLA-B27 is associated with longer diagnosis delay time and higher frequency of psoriasis.

## Introduction

Axial spondyloarthritis (SpA) describes a heterogeneous group of rheumatic diseases characterized by axial skeleton involvement. It can also involve other sites such as peripheral arthritis, enthesitis, dactylitis, and extra-articular manifestations as uveitis, psoriasis, and inflammatory bowel disease (IBD). The term axial spondyloarthritis affects patients with both non-radiographic and radiographic axial spondyloarthritis, which is also termed ankylosing spondylitis (AS) (1).

Investigators have estimated that HLA-B27 in the major histocompatibility complex (MHC) locus contributes to ~20.1% of AS heritability (2). HLA-B27 is found in 74–89% of patients with either non-radiographic axial spondyloarthritis or ankylosing spondylitis by GESPIC (3) and Herne cohort (4). Moreover, previous studies suggest a relationship between HLA-B27 and clinical manifestations. HLA-B27 is also known to be associated with earlier age of axial SpA onset (3, 5), increased severity and persistence of MRI-demonstrated inflammation at the sacroiliac joints (SIJ) and lumbar spine in early low back pain (IBP) (6), and anterior uveitis in SpA patients (5, 7). However, the clinical characteristics of axial SpA patients related to HLA-B27 presence have not been determined in a large cohort of Chinese patients to date. Since previous studies focused mainly on its association with AS, the exact role of HLA-B27 in axial SpA remains unknown in China. Furthermore, the distribution of HLA-B27 population is various. Higher rates of HLA-B27 were found in ax-SpA patients of North America and Western Europe (80–95%) (8) than in Japan and Arab countries (41–84%) (9, 10). Meanwhile, the data for China is limited.

This study aimed to evaluate the HLA-B27 influence on the clinical expression of axial SpA patients. Hence, we reviewed data from the China Spondyloarthritis (ChinSpA) registry database, which includes more than 4,000 axial SpA patients.

## Materials and Methods

### Patients and Study Design

The ChinSpA registry is a longitudinal observational study exploring the clinical features, predictors of SpA activity, and severity of Chinese patients. It is available through a computerized internet database accessible to all participating physician members. The data of this study is from ChinSpA registry. The inclusion criteria of this study were: 1) Patients were required to have a clinical diagnosis of axSpA by a rheumatologist, and fulfilled the classification criteria of Assessment of SpondyloArthritis international Society (ASAS) (11). AxSpA can be subclassified as radiographic axSpA (r-axSpA) such as ankylosing spondylitis or non-radiographic axSpA (nr-axSpA) according to the presence or absence of sacroiliac joint damage on X ray (11). The ASAS classification of axSpA relies either on sacroiliitis on imaging plus one SpA feature (imaging arm) or HLA-B27 antigen plus two SpA features (clinical arm), in a patient with chronic low back pain and age at onset of <45 years. 2) Patients who were at least 18 years old. 3) If the information of the HLA-B27 status were available. It collected baseline data for patients enrolled from departments of Rheumatology in 225 hospitals from 31 provinces in China. Patient recruitment was between August 2018 and May 2020. The study protocol was in accordance with the guidelines of the Helsinki Declaration and was approved by the ethical committee of Peking Union Medical College Hospital and all the other participant hospitals. Written informed consent was obtained from all participants.

All clinical parameters were examined by the rheumatologists in the registry. Patients were interviewed for baseline characteristics which included gender, age at enrollment, age at onset, age at diagnosis, relevant family history, presence of SpA features, extra-articular manifestations, disease activity, medication including use of non-steroidal anti-inflammatory drugs (NSAIDs), disease-modifying antirheumatic drugs (DMARDs), and biologic treatment. Most patients could provide clear information on past clinical manifestation such as age at onset, past peripheral arthritis, and past dactylitis.

Family history was defined as whether the subject had aony first-degree relatives (mother, father, sisters, brothers, and children) or second-degree (maternal and paternal grandparents, aunts, uncles, nieces, and nephews) relatives with history of any of the following: (1) AS; (2) psoriasis; (3) acute uveitis; (4) reactive arthritis; and (5) IBD (11).

A 44-joints count has been proposed to measure peripheral joint involvement, which includes the sternoclavicular joints, acromioclavicular joints, shoulders, elbows, wrists, knees, ankles, MCP, and MTP joints, and PIP joints of the hands. Peripheral arthritis was defined as the presence of swelling in at least one peripheral joint.

The Maastricht Ankylosing Spondylitis Enthesitis Score has been proposed to measure enthesitis involvement (12), which includes costochondral 1 right/left, costochondral 7 right/left, spina iliaca anterior superior right/left, crista iliaca right/left, spina iliaca posterior right/left, processus spinosus L5, achilles tendon, and proximal insertion right/left.

Laboratory tests included C-reactive protein (CRP) and erythrocyte sedimentation rate (ESR) and the HLA-B27 status. Disease activity assessment such as the Bath Ankylosing Spondylitis Disease Activity Index (BASDAI) (13), the Bath Ankylosing Spondylitis Functional Index (BASFI) (14), patient global assessment (PGA), spinal pain, and night back pain were performed. The Ankylosing Spondylitis Disease Activity Score (ASDAS) was calculated with CRP (15).

### Statistical Analyses

All quantitative data were described as mean and standard deviation for the normal distribution or median and interquartile range (IQR) for the non-normal distribution. Additionally, unpaired *T*-tests or Wilcoxon rank-sum tests were performed, respectively, to compare both groups. Frequencies and percentages were used for qualifying data and were compared by chi-square test or Fisher's exact tests.

All statistical analyses were performed using SAS 9.4 (SAS Institute, Cary, NC, USA), and significance was set at *p* < 0.05. Statistically significant variables according to univariate analysis were considered in the multiple logistic regression models. Stepwise selection method was adopted for estimating parameters using *p* = 0.10 as a criterion for variable entry and *p* = 0.05 as a criterion for variable stay in the model. The associations were represented by odds ratio (OR) and 95% confidence interval.

## Results

Amon total 4,444 ax-Spa patients, 4,131 (93.0%) were tested for HLA-B27. Altogether, 4,131 patients were included in this study; of those, 3,695 (89.4%) with HLA-B27 and 436 without HLA-B27 were analyzed. 609 (14.7%) patients fulfilled the imaging arm, 207 (5.0%) fulfilled the clinical arm and 3,315 (80.3%) fulfilled the both clinical and imaging arms. 73.1% of all axial patients were male, and the mean disease duration was 6.69 ± 7.06 y. The comparison of characteristics between patients with and without HLA-B27 is shown in Table 1.

**Table 1:** Baseline demographics, clinical features and treatments in Ax-SpA patients with and without HLA-B27.

**Variables**	**With HLA-B27 *N* = 3,695**	**Without HLA-B27 *N* = 436**	***p*-value**
Male, *n* (%)	2,774 (75.1)	247 (56.7)	<0.001
Age at enrollment (years), mean ± SD	33.22 ± 10.68	37.35 ± 13.0	<0.001
Age of onset (years), mean ± SD	26.21 ± 9.45	31.29 ± 12.26	<0.001
Age at diagnosis, years (mean ± SD)	29.57 ± 10.46	34.98 ± 13.52	<0.001
Disease duration (years), median (Q1, Q3)	5 (2, 10)	3 (1, 8)	<0.001
Diagnosis delay time (months), median (Q1, Q3)	12 (1, 48)	17.5 (2, 58.5)	0.034
Family history, *n* (%)	899 (24.3)	50 (11.5)	<0.001
Peripheral manifestations			
Current or past peripheral arthritis, *n* (%)	1,321 (35.8)	142 (32.6)	0.189
Current peripheral arthritis, *n* (%)	999 (27.0)	158 (36.2)	0.001
Current or past heel pain *n* (%)	832 (22.5)	114 (26.2)	0.088
Current enthesitis, *n* (%)	2,386 (64.6)	297 (68.1)	0.146
Current or past dactylitis, *n* (%)	216 (6.3)	40 (9.7)	0.010
Current hip joint involvement, *n* (%)	1,135 (33.2)	106 (25.7)	0.002
Extra-articular manifestations			
Current or past uveitis, *n* (%)	412 (11.2)	22 (5.1)	<0.001
Current or past psoriasis, *n* (%)	27 (0.7)	11 (2.5)	<0.001
Current or past inflammatory bowel disease, *n* (%)	55 (1.5)	11 (2.5)	0.103
BASDAI, mean ± SD	3.68 ± 2.22	3.66 ± 2.12	0.970
BASFI, median (Q1, Q3)	2.1 (0.6, 4.3)	1.9 (0.6, 3.85)	0.129
CRP, mg/L, median (Q1, Q3)	10 (3.11, 24.5)	5 (1.42, 17.25)	<0.001
ASDAS-CRP, mean ± SD	2.69 ± 1.23	2.42 ± 1.21	<0.001
Good response to NSAIDs, n (%)	2,728 (79.7)	310 (75.1)	0.027
NSAIDs	2,298 (67.2)	306 (74.1)	0.004
Biologics	1,741(50.9)	141 (34.1)	<0.001
DMARDs	2,073 (60.6)	268 (64.9)	0.091

*Ax-SpA, Axial spondyloarthritis; BASDAI, Bath Ankylosing Spondylitis Disease Activity Index; BASFI, Bath Ankylosing Spondylitis Functional Index; CRP, C-reactive protein; ESR, erythrocyte sedimentation rate; ASDAS, Ankylosing Spondylitis Disease Activity Score; NSAIDs, non-steroidal anti-inflammatory drugs; HLA-B27, human leukocyte antigen B27*.

The proportion of male is higher in patients with HLA-B27 than in patients without HLA-B27 (75.1 vs. 56.7%, *p* < 0.001). Patients with HLA-B27 showed younger age at onset (*p* < 0.001), younger age at diagnosis (*p* < 0.001), longer disease duration (*p* < 0.001), and shorter diagnosis delay time (*p* = 0.034) than patients without HLA-B27. Moreover, patients with HLA-B27 showed a greater prevalence of family history than patients without HLA-B27 (24.3 vs. 11.5%, *p* < 0.001) (Table 1).

Compared to the patients without HLA-B27, those with HLA-B27 had a significantly higher likelihood of hip joint involvement (33.2 vs. 25.7%, *p* = 0.002), but a lower likelihood of peripheral arthritis (27.0 vs. 36.2%, *p* < 0.001) and dactylitis (6.3 vs. 9.7%, *p* = 0.01) at enrollment. However, no differences were observed between both groups regarding the prevalence of current or past peripheral arthritis. For extra-articular manifestations, patients with HLA-B27 showed a lower prevalence of psoriasis (0.7 vs. 2.5%, *p* < 0.001), a higher prevalence of uveitis (11.2 vs. 5.1%, *p* < 0.001) than patients without HLA-B27. Furthermore, patients with HLA-B27 expressed a higher disease activity measured by CRP (*p* < 0.001) and ASDAS-CRP (*p* < 0.001). However, this study did not find any statistical differences regarding the BASDAI and BASFI. For treatment, patients with HLA-B27 showed a greater prevalence with biologic treatment (50.9 vs. 34.1%, *p* < 0.001), but a lower prevalence with NSAIDs (67.2 vs. 74.1%, *p* = 0.004) than patients without HLA-B27 at enrollment (Table 1).

In the multivariate analysis, men (*p* < 0.001), younger age (*p* < 0.001), disease duration >3 years (*p* < 0.001), family history (*p* < 0.001), uveitis (*p* < 0.001), higher ASDAS-CRP (*p* < 0.001), and on biologic treatment (*p* < 0.001) were the main variables that are independently related to HLA-B27 presence, whereas diagnosis delay time > 36 months (*p* < 0.001), and psoriasis (*p* < 0.001) were independently related to HLA-B27 absence (Table 2).

**Table 2:** Multivariate logistic regression analysis of clinical characteristics of Ax-SpA patients based on the HLA-B27 status.

	**Univariate logistic regression**		**Stepwise logistic regression**	
Variables^*^	**OR (95% CI)**	***P*-value**	**OR_**adj**_ (95% CI)**	***P*-value**
Male (ref=male)	0.43 (0.35–0.53)	<0.001	0.58 (0.46–0.72)	<0.001
Age/years	0.97(0.96–0.98)	<0.001	0.97 (0.96–0.98)	<0.001
Disease duration > 3 years (ref= “ < =3years”)	1.45 (1.19–1.76)	<0.001	1.84 (1.40–2.43)	<0.001
Diagnosis delay time (<36 vs. <3 months)	0.79(0.62–1.02)	0.625	0.76 (0.58–1.00)	0.678
Diagnosis delay time (≥36 vs. <3 months)	0.70 (0.54–0.90)	0.021	0.52 (0.38–0.71)	<0.001
Family history (ref=none)	2.48 (1.83–3.36)	<0.001	2.54 (1.84–3.52)	<0.001
Current peripheral arthritis (ref=none)	0.65(0.53–0.80)	<0.001	0.61 (0.48–0.77)	<0.001
Dactylitis (ref=none)	0.63 (0.45–0.89)	0.008	N.A.	N.A.
Hip joint involvement (ref=none)	1.42 (1.14–1.78)	0.002	N.A.	N.A.
Psoriasis (ref=none)	0.28 (0.14–0.58)	<0.001	0.18 (0.08–0.40)	<0.001
Uveitis (ref=none)	2.36 (1.52–3.67)	<0.001	2.88 (1.81–4.60)	<0.001
ASDAS-CRP	1.20 (1.10–1.30)	<0.001	1.31 (1.19–1.44)	<0.001
NSAIDs (ref=none)	0.72 (0.57–0.90)	0.005	N.A.	N.A.
Good response to NSAIDs (ref=none)	1.33 (1.05–1.67)	0.016	N.A.	N.A.
Biologics (ref=none)	2.00 (1.61–2.48)	<0.001	1.75 (1.40–2.18)	<0.001

* *After fully considering collinearity of variables and clinical interpretability, all of these variables were used in stepwise logistic regression, while dactylitis, Hip joint involvement, NSAIDs, and Good response to NSAIDs were not included in final multiple model*.

*Age and ASDAS-CRP were added as a continuous variable; the statistical method of trisection was used for Diagnosis delay time, and other categorical variables are dichotomous*.

*Ax-SpA, Axial spondyloarthritis; HLA-B27, human leukocyte antigen B27; ASDAS, Ankylosing Spondylitis Disease Activity Score; CRP, C-reactive protein; OR, odds ratio; CI, confidence interval*.

## Discussion

Here, we analyzed the clinical characteristics of axial SpA patients according to the HLA-B27 status based on a large dataset of China. We observed a higher proportion of the male sex, younger age, longer disease duration, and family history in axial SpA patients with HLA-B27 than in those without HLA-B27. Moreover, patients with HLA-B27 showed a higher prevalence of uveitis and higher disease activity measured by ASDAS-CRP than in those without HLA-B27. On the other hand, absence of HLA-B27 is associated with longer diagnosis delay time and higher frequency of psoriasis.

In all patients with axial SpA in our study, the prevalence of HLA-B27 presence was 89.4%. A systemic review reported that the prevalence of HLA-B27 in axSpA patients fulfilling any of the contemporary criteria [New York (NY), Amor, ESSG, ASAS] ranged from 26.2 to 91% (9, 11). In addition, in AS patients (radiographic axSpA), the prevalence of HLA-B27 presence was 94.8, 83, 71, and 79%, in South Korea, Europe, Latin America and Canada, respectively (16–19). The differences possibly due to genetic backgrounds. Moreover, 73.1% of all patients with axial SpA were male in this study. Similar results were also described in the REGISPONDER, SPARCC, and PSOAS registries (17, 19). However, in a Korean study, a higher proportion of the male sex (88.2%) was reported in 830 patients with radiographic axial SpA, whereas 58.6% were male in a French study (16, 20). This study found a higher prevalence of male axSpA patients with HLA-B27, which is in accordance with that previously published in another Chinese research on radiographic SpA, and also similar in a DESIR cohort conducted in patients representing early forms of the whole spectrum of axial SpA (20, 21). However, several other studies did not note any differences (16, 22–24), which may be explained by considering geographic and genetic factors.

We observed that the mean age at onset of axial SpA was 5 years earlier in patients with HLA-B27 than in those without HLA-B27 (26 vs. 31 years). The multivariate analysis result showed that younger age was independently related to HLA-B27 presence, similarly with previous studies (5, 16, 17, 20, 24, 25). This study also showed that the diagnosis delay time was significantly longer in patients with HLA-B27 negative compared to patients with HLA-B27 positive, suggesting that HLA-B27 is helpful for the diagnosis of axSpA at early phase. Additionally, our results showed that axial SpA patients with HLA-B27 had greater family aggregation than in those without HLA-B27, which is consistent with previous studies in radiographic axial SpA and in early forms of SpA patients from DESIR (17, 20, 21). Furthermore, the finding that 11.5% of patients without HLA-B27 had family history was in accordance with previous researches, supporting the existence of unknown genetic factors other than HLA-B27 that has a role in the familial aggregation of the disease (17, 20, 21).

There was no difference in musculoskeletal manifestations such as peripheral arthritis (current or past), heel pain (current or past), and dactylitis (current or past) between both groups, except a lower prevalence of current peripheral arthritis was found among patients with HLA-B27 at enrollment. In previous studies, the findings regarding the comparison of musculoskeletal manifestations between both groups were inconsistent. In another Chinese research (21), in accordance with ours, the authors found no differences in musculoskeletal manifestations between both groups; whereas in the DESIR cohort (20), the prevalence of present peripheral arthritis was lower in patients with HLA-B27 than in those without HLA-B27. Given that current peripheral arthritis was associated with disease activity, the discrepancy in results on the manifestations may be explained by the different statuses of disease activity upon collection of patient information.

A meta-analysis showed that the prevalence of uveitis in AS patients was associated with disease duration and ranged from 17.4% to 38.5% in patients with a mean disease duration of <10 and >20 years (26). Here, the prevalence of uveitis was 10.5% in axial SpA patients with a mean disease duration of 6.9 years. Different geographical areas may explain part of the variation. Furthermore, our result that uveitis was independently related to HLA-B27 presence was in accordance with previous studies (27, 28). In contrast, another previous study showed that in patients with uveitis and HLA-B27 presence, the risk of developing SpA is significantly higher than in uveitis patients without HLA-B27 (29). The prevalence of psoriasis and IBD of this study was 0.9 and 1.6%, respectively, which were lower than those reported in previous studies (26). The discrepancy of prevalence may be due to differences in disease duration and geographical area. Moreover, we did not observe any differences in IBD presence between both groups. Previous study reported a lower prevalence of IBD among patients with HLA-B27 (17). However, no clear evidence has been found to prove that the risk of developing IBD or subclinical IBD is higher in axSpA patients with HLA-B27 (8). Additionally, this study showed that the prevalence of psoriasis was lower in patients with HLA-B27 than in those without HLA-B27, which was consistent with previous studies (17, 20). Psoriasis being negatively associated with HLA-B27 presence may be due to the selection bias of patients without HLA-B27 according to the Amor criteria as they require more extra-articular features in order to be diagnosed with SpA (20).

Regarding disease severity, the results reported were inconsistent. Some studies reported similar BASDAI and BASFI scores in patients with and without HLA-B27 (16, 24, 25), whereas other studies showed worse BASDAI and BASFI scores in patients without HLA-B27 (17). Here, we observed no differences in BASDAI and BASFI scores in both groups but a higher level of ASDAS-CRP in patients with HLA-B27. However, a positive correlation between presence of HLA-B27 and high ASDAS-CRP was not previously confirmed (20, 30, 31). The results regarding disease severity depend on patient status and treatment upon data collection. To confirm whether HLA-B27 influences the disease severity, longitudinal studies using average scores of the BASDAI and BASFI should be conducted in the future.

For treatment, no difference was observed between both groups regarding satisfactory response to NSAIDs in multivariate analysis at enrollment. However, no similar research has been found in the past. Previous studies showed that patients with HLA-B27 show a better clinical response to Tumor necrosis factor inhibitor (TNF) inhibitors (32–34). This was a cross-sectional study and did not evaluate the therapeutic effect. Furthermore, the results showed that patients with HLA-B27 used more biological agents than did those without HLA-B27 at enrollment, which may be because of higher disease activity of those with HLA-B27 as discussed earlier.

This study has some limitations that should be noted. First, this is a cross-sectional study, and status of disease activity only represented the moment assessing the disease activity. Analyzing whether the HLA-B27 status influences the disease severity using a mean of BASDAI and BASFI scores in a longitudinal cohort may yield more accurate results. We did not analyze the treatment response using TNF alpha inhibitors, which also need a longitudinal cohort. Second, the mean disease duration of patients in this study was 6.9 years. Since some clinical manifestations such as uveitis were associated with disease duration, the results of patients with longer disease duration must be interpreted with caution. Third, only patients with available HLA-B27 were included in the analysis. The patients without any available HLA-B27 might have different characteristics, which may influence the results. Lastly, we did not analyze the influence of HLA-B27 on structural damage.

## Conclusion

In summary, this is an extensive study assessing the influence of the HLA-B27 status on axial SpA in the largest Chinese cohort. Our study confirms HLA-B27 is associated, in axial SpA patients, with the male sex, younger age, longer disease duration, greater family aggregation, and higher frequency of uveitis. However, we don't support an association between the HLA-B27 status and any musculoskeletal manifestations. Meanwhile, absence of HLA-B27 is associated with longer diagnosis delay time and higher frequency of psoriasis.

## Data Availability Statement

The raw data supporting the conclusions of this article will be made available by the authors, without undue reservation.

## Ethics Statement

The studies involving human participants were reviewed and approved by ethical committee of Peking Union Medical College Hospital and all the other participant hospitals. The patients/participants provided their written informed consent to participate in this study.

## Author Contributions

SZ wrote the paper. JS and XZ were involved in the protocol development, study design, supervision of the data collection, critical revision, reviewing of the report, and quality checking. ML and LP reviewed the paper. YW performed the statistical analysis. ZW, JX, MY, LW, CZ, XD, QL, and WF contributed to the data collection. All authors read and approved the final manuscript.

## Conflict of Interest

The authors declare that the research was conducted in the absence of any commercial or financial relationships that could be construed as a potential conflict of interest.
